# The power of mumps virus: Matrix protein activates apoptotic pathways in human colorectal cell lines

**DOI:** 10.1371/journal.pone.0295819

**Published:** 2023-12-13

**Authors:** Solmaz Morovati, Ali Mohammadi, Ramin Masoudi, Amir Ali Heidari, Mehdi Asad Sangabi

**Affiliations:** 1 Department of Pathobiology, Division of Biotechnology, School of Veterinary Medicine, Shiraz University, Shiraz, Iran; 2 Department of Pathobiology, Division of Virology, School of Veterinary Medicine, Shiraz University, Shiraz, Iran; 3 Department of Clinical Sciences, Division of Aquatic Animal Health and Diseases, School of Veterinary Medicine, Shiraz University, Shiraz, Iran; University of Rajshahi, BANGLADESH

## Abstract

New therapeutic approaches can significantly impact the control of colorectal cancer (CRC), which is increasing worldwide. In this study, we investigated the potential of targeting viral proteins to combat cancer cells. Specifically, we examined the anticancer potential of the matrix (M) protein of the mumps virus Hoshino strain in SW480 CRC cell lines. To begin, we individually transfected SW480 cells with pcDNA3 plasmids containing the mumps virus M gene. We then investigated the percentage of cell death, caspase activity, and the expression levels of genes involved in apoptosis pathways. Following this, we performed bioinformatics analysis on the M protein to identify any similarities with Bcl-2 family members and their viral homologs. Our diagnostic methods showed that treatment with the mumps M protein induced apoptosis and upregulated the expression and activity of pro-apoptotic proteins in SW480 CRC cells compared to the control and vector groups. Based on our bioinformatics studies, we proposed that the BH3 motif in the M protein may trigger apoptosis in CRC cells by interacting with cellular Bax. Overall, our study showed for the first time that the mumps virus M protein could be considered as a targeted treatment for CRC by inducing apoptotic pathways.

## Introduction

Metastatic cancer, a condition that is predominantly resistant to current treatment options, necessitates the exploration and development of innovative therapeutic approaches [1–3]. Colorectal cancer (CRC) is ranked 3rd with a detection rate of about (6.1%) and ranks second in mortality (9.2%). Conventional cancer treatment methods, such as chemotherapy and radiotherapy, are insufficient and are associated with increased side effects and damage to thenormal body tissues. Therefore, in recent years, with the development of new methods such as onco-virotherapy, efforts have been made to enhance the effectiveness of traditional treatment methods as well as novel immunotherapies. For this purpose, various members of *Adenoviridae* [4], *Coronaviridae* [5], *Herpesviridae* [6], *Picornaviridae* [7], *Retroviridae*, *Reoviridae* [8], *Poxviridae* [9], *Paramyxoviridae* [10–13], and *Rhabdoviridae* [14] have been investigated for their oncolytic properties and modified in preclinical and clinical trials.

Cellular Bcl-2 (cBcl-2) family proteins contain one or more Bcl-2 homology (BH) domains, which are key mediators of cellular apoptotic pathways. The BCL-2 family protein interaction with one another and other proteins enhances or inhibits apoptosis in host cells. This process depends on the BH3 or transmembrane (TM) regions in one protein and a hydrophobic pocket formed by BH1 to BH3 regions in other proteins that affects mitochondrial membrane permeabilization (MMP) [15]. Some studies have discovered homologs of Bcl-2 in viral genomes that influence different aspects of viral pathogenesis [16–26]. Targeting viral death proteins may have clinical significance in treating tumor cells. These proteins can be used to combat cancer cells by activating anti-tumor immunity and triggering apoptosis.

Mumps is one of several infectious diseases in children that can be controlled by vaccination [26]. The causative agent is an enveloped particle from the *Paramyxoviridae* family that contains a non-segmented negative-strand RNA molecule of 15,384 nucleotides. The encapsidated genome contains seven tandemly linked transcription units (Nucleo, V/phospho-/I proteins, matrix (M), fusion, small hydrophobic, hemagglutinin-neuraminidase, and large proteins) [18]. In addition to other paramyxoviruses (measles virus, Newcastle disease virus (NDV), peste des petits ruminants virus (PPRV), and canine distemper virus), mumps virus has captured the interest of scientists in treating human malignancies [26].

Although the destructive effects of mumps virus on CRC cells have not yet been studied, this property has been reported in other tumor cells [18,27–32]. However, most studies have focused on the oncolytic effects of whole mumps virus particles. To our knowledge, only one study has addressed the role of the V protein in the Urabe AM9 vaccine strain in the apoptotic pathway [33]. Indeed, the oncolytic activity of the Hoshino vaccine lineage has been confirmed in fibrosarcoma and cervical cancer cell lines [18].

Here, for the first time, we studied the cytotoxicity of the mumps virus Hoshino commercial vaccine strain M protein in human SW480 CRC cells. We investigated the M sequence of the mumps virus for any potential BH domains or connections to their counterparts in the Bcl-2 family members or other viral homologs. Using wet-lab experimental analysis, we estimated the viability and apoptosis rate of SW480 CRC cell lines transfected with pcDNA3 plasmid encoding the M protein. Then, the contribution and activity of apoptosis-related proteins in SW480 cancer cell death were examined.

## Materials and methods

### Cell culture

The Pasteur Institute, Iran, provided the SW480 human CRC cell line (NCBICode#C506), which was transferred to a sterile environment and placed in an incubator at 37°C and 5% CO2. Fresh culture complete growth medium DMEM/F-12 (Sigma-Aldrich, St. Louis, Mo, USA; Catalog #D8437) with high glucose, 10% FBS (Bio-Idea, Iran; Catalog #BI-1201), and 1% penicillin/streptomycin was used for cell culture. 0.25% (w/v) trypsin-EDTA (Sigma-Aldrich, St. Louis, Mo, USA; Catalog #T2610) were used to separate the cell monolayer from the bottom of the flasks for cell culture. Isolated cells were counted usingtrypan blue (BioSera, France; Catalog #LM-T1708), and a hemocytometer.

### RNA extraction and RT-PCR

Before RNA extraction, the viral vaccine strain (Razi Vaccine and Serum Research Institute, Iran) was cultured in Vero cells (NCBICode#C101) to obtain the desired amount of genome. The procedure for virus isolation has been described previously [5]. The Vero cells were monitored daily for CPE formation and after seven days the viruses were harvested using repeated freeze-thaw cycles. The attached cells were also separated using 0.25% (w/v) trypsin-EDTA (Sigma-Aldrich, St. Louis, Mo, USA; Catalog #T2610). The cells were then diluted in a complete growth medium. Then, the homogenized sample was mixed with 1 ml ice-cold RNX TM–PLUS solution (CinnaGen, Iran; Catalog #RN7713C). Next, 200 μl of chloroform was added, the aqueous phase was transferred to a new RNase-free tube, and an equal volume of isopropanol was added. After centrifugation, the supernatant was discarded, and the cells were washed with 1 ml of 75% ethanol. Finally, the pellet was dissolved in 50 μl of DEPC-treated water.

Total RNA 1 μg, 2x Reaction Buffer 10 μl, 10 mM dNTP Mixture 2 μl, 10x random hexamer 2 μl, 20x Enzyme Solution, and DEPC-treated water were pipetted into RNase-free tubes for a total reaction volume of 20 μl. The tubes were then incubated for 10 min at 25°C and 50 min at 60°C. RT inactivation was achieved by heating for 5 min at 80°C. It was cooled on ice. PCR conditions were as follows: 95°C for 2 min, followed by 95°C for 30 s, 31 cycles of 56.2°C for 30 s, and 72°C for 2 min. The final extension was performed at 72°C for 20 min. Finally, the amplified products were gel-purified using 1% agarose gel (CinnaGen, Iran; Catalog #EP5052). cDNA synthesis kits were obtained from (ADDBIO, Korea; Catalog # KCAS37).

### Plasmid construction

A primer set with a sequence of forward 5′-CTACTCGAGACCATGGCCGGATCACAGATC-3′ and reverse 5′-CTGGGGCCCTCACAGGTTGCTCATTGAGGC-3′ was designed to amplify the coding sequence of the mumps virus *M* gene. The sequences of XhoI and ApaI restriction sites and the Kozak sequence were also introduced into the *M* gene sequence by designing the primers. The *M* genes were first subcloned into pTZ57R/T plasmids using a TA cloning vector kit (Thermo Scientific InsTAclone PCR Cloning Kit, USA; Catalog #K1213), according to the manufacturer’s instructions. For Xhol and Apal Cloning, the purified M fragment was cloned into the pcDNA™3.1/Hygro(+) expression vector. A plasmid extraction kit (Bio Basic, Canada; Catalog #BS413), DNA extraction kit from the gel (Bio Basic, Canada; Catalog #BS654), XhoI enzyme (Thermo Scientific, USA; Catalog #ER0691), and ApaI enzyme (Thermo Scientific, USA; Catalog #ER1411) were prepared. Finally, the plasmids were transformed into E. coli DH5α cells (Pasteur Institute, Iran; ATCC #25922) using the calcium chloride heat-shock method, as described previously [34].

### Study design

The study groups were categorized as follows:

Control: Untreated SW480 cancer cells

pcDNA3-Matrix (pcDNA3-M): pcDNA3 plasmid containing the *matrix* gene of mumps virus.

Vector: pcDNA^TM^3.1/Hygro^(+)^ mammalian expression vector (without foreign *matrix* gene).

### Cell proliferation assay (MTT)

SW480 cancer cells were cultured in a special cell culture plate and treated with pcDNA3-M recombinant plasmid and pcDNA3 vector without any foreign gene. The cells were incubated for 48, 72, and 96 h. The cells were transfected with a mixture of 7.8 μl DNA/CaCl2 (a total of 0.3 μg/well of pcDNA3/ pcDNA3-M plasmid DNA) and 7.8μl 2XHBS. Then, the reaction was followed in the presence of 10 μL MTT (12 Mm) (Sigma-Aldrich, St. Louis, Mo, USA, Catalog #11465007001) for 4 h at 37°C to reduce the colorless tetrazolium dye MTT to insoluble formazan, which is purple in color. The formazan was dissolved in 50 μL of DMSO (Sigma-Aldrich, St. Louis, Mo, USA, Catalog # C6295), and the optical density of each well was measured at 570 nm. Cell viability (%)was calculated using the GraphPad Prism (version 9). The experiment was repeated three times, with similar results.

### Flow cytometry

Flow cytometry was performed to determine the percentage of apoptotic SW480 cells. The cultured cells were treated with the *M* genes of the mumps virus and pcDNA3 for 48, 72, and 96 h. An apoptosis and necrosis quantitation kit was used to stain apoptotic cells with green fluorescence and necrotic cells with red fluorescence for examination by flow cytometry. The transfection medium was formulated as follows: 2.5μg DNA total; cells in 1.12 ml medium; 62.5μl total volume of DNA/CaCl2 mix (with 8μl 2M CaCl2); 62.5μl 2XHBS. The cells were rinsed twice with cold phosphate-buffer (PBS) and suspended in 1X binding buffer (5×105 cells/well). The cells were stained sequentially with annexin V-FITC (green fluorescence) (Padza Padtan Pajhooh; Iran; Catalog #K-01) and propidium iodide (red fluorescence) and analyzed using the FlowJosoftware (version 10). The experiments were performed in triplicates.

### Gene expression analysis by Real-Time PCR

The specific primers for GAPDH (as internal control), p53, Bax (Bcl-2-associated X protein), Bcl-2 (B-cell lymphoma 2), caspase 8, and caspase 9 used in this study have been described previously [12]. The expression of the genes was studied 72 h after treatment using the transfection mixture as follows: (3 μg DNA total; cells in 2.25 ml medium; 125μl total volume of DNA/ CaCl2 mix (with 16μl 2M CaCl2); 125μl 2XHBS). Next, RNA was extracted and reverse-transcribed according to the manufacturer’s protocol. The PCR reaction components consisted of Real Plus and 2X SYBR Green PCR Master mix (Amplicon), Primer A 10μM (0.5 μl (0.25–2.5 μl) Vol./reaction, 0.1 μM (0.05–0.5 μM)), Primer B 10μM (0.5 μl (0.25–2.5 μl) Vol./reaction, 0.1 μM (0.05–0.5 μM), PCR-grade H2O (10.5 μl Vol./reaction), Template DNA (1μl) and a total volume of 25 μl (1 cycle, 15 min cycle duration, 95°C), (40 cycles, 30 s duration of cycle at 95°C), and (30 s duration of cycle at 61.5°C). This assay was performed three times for each treatment group.

### Caspase activation assay

According to the manufacturer’s instructions (Kiazist Life Sciences, Iran; Catalog #22701), SW480 cancer cells were treated with recombinant plasmids for 72 h after transfection. SW480 cells at a density of 5×10^5^ cells were lysed with 500 μL caspase lysis buffer and incubated for 20 min at 4°C. The solution was processed by centrifugation at12000 rpm for 15 min, and the supernatant was stored at -80°C for downstream processes. To obtain a standard concentration of 500 μM, 50 μL of the standard vial was diluted with 450 μL of caspase lysis buffer. Six standard solutions of different concentrations (0, 10, 20, 30, 40, and 50 μM) were prepared. Fifty microliters of each sample, positive control, and standard were added to the ELISA plate. Then 55.5 μL of the solution containing caspase buffer: 50 μL, dithiothreitol: 0.5 μL, and caspase substrate: 5 μL was added to each well and incubated for 2 h at 37°C. The assay was performed twice in each group.

Caspase 3 and caspase 7 activities at an excitation wavelength of 405 nm were measured using **Eq 1.**


$$Caspase\;Activity=\frac{Sample\;concentration\;(\mu mol)\times 105.5}{Incubation\;time\;(minute)\times 50}\times dilution\;factor$$ Eq 1

### Protein sequence and phylogenetic analysis

Protein sequence analysis was performed to identify the BH motifs in the mumps virus M protein sequence. Clustal W software [28] was used to perform multiple alignments of the sequences of the M protein and Bcl-2 family members and their viral homologs. The SWISS-MODEL repository (https://swissmodel.expasy.org/repository) was used to compare protein secondary structures. UCSF Chimera software (version 1.10.2) [29] displayed t 3D structures. The HPEPDOCK 2.0 web program (http://huanglab.phys.hust.edu.cn/hpepdock/) was used for the docking analysis.

To examine the evolutionary links between viral proteins with BH domains, as well as Bcl-2 family members, a phylogenetic tree was generated using the neighbor-joining method. Protein sequences were compared, and similarities and differences were assessed using MEGA software version 7.22.

### Statistical analysis

Data were expressed as mean ± SD, and graphs were constructed using the GraphPad Prism program (version 9). Data were statistically analyzed using analysis of variance (Two-way ANOVA) or t-test (paired) followed by a post-Tukey test, and a *p*-value≤0.05 was defined as a significant difference.

## Results

### The M protein of mumps virus significantly decreased survival of the SW480 cells compared to the control group

**Fig 1:**
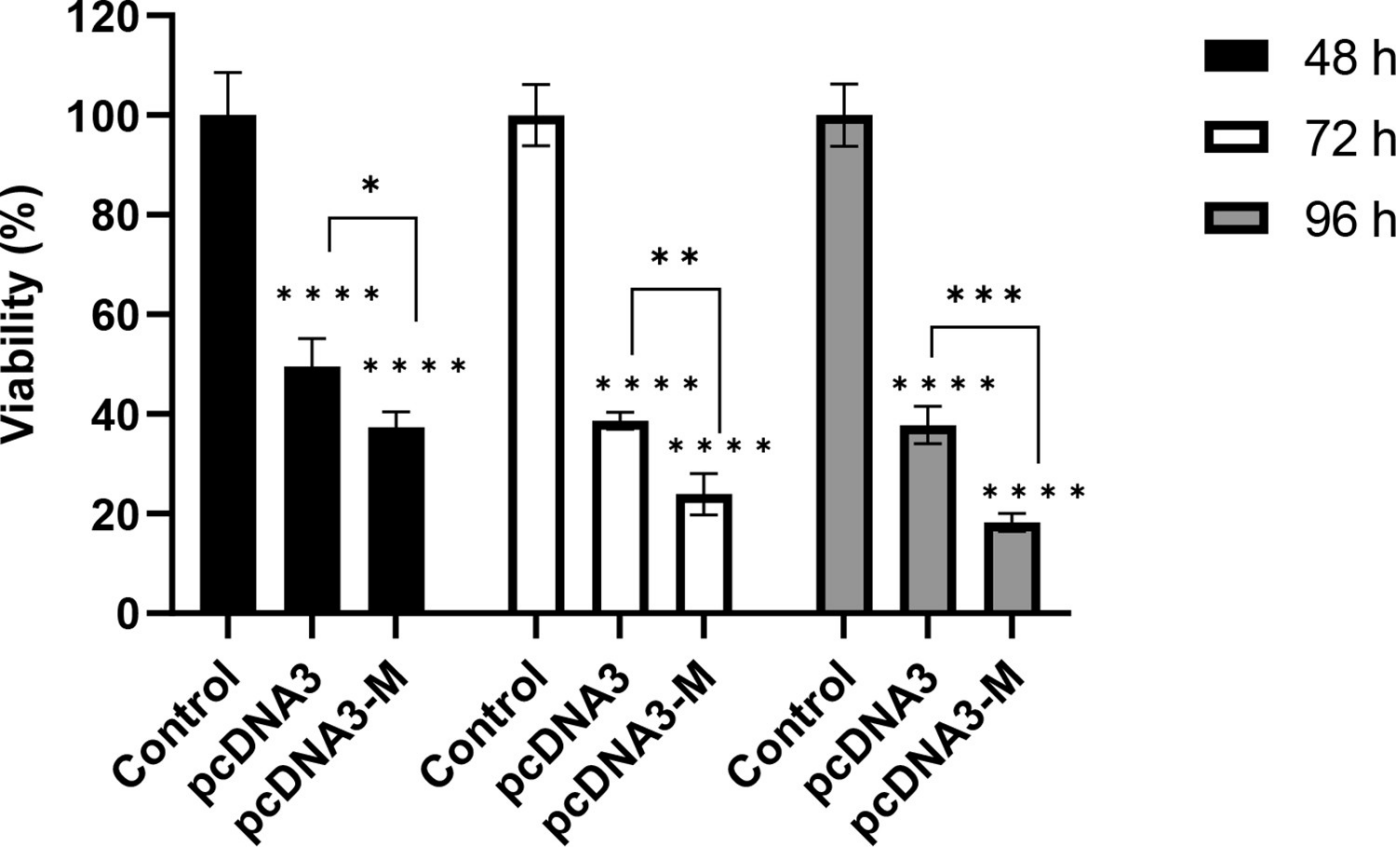
The cytotoxic effects of pcDNA3 and Mumps M protein on SW480 cells. The viability of the cancer cells in each group was measured using the MTT assay after 48, 72, and 96 h. The control group consisted of the uninfected cells. The results showed that cell viability in the group treated with recombinant pcDNA3-M differed significantly from that in control and vector (pcDNA3) groups. Data represent the mean ± standard deviation (n = 4). For all charts, *: *p<*0.01; **: *p<*0.01; ***: *p<*0.001; ****: *p<*0.0001.

Cell cytotoxicity assay (MTT) was used to evaluate the percentage of SW480 cell viability after treatment with pcDNA3 and pcDNA3-M for 48, 72, and 96 h (Fig 1). The treatment group with the recombinant plasmid harboring the M gene of mumps virus (pcDNA3-M) showed a decrease in viability percentage of SW480 cancer cells compared to the control group (untreated) (*p<*0.0001) and pcDNA3 vector (*p<*0.05). Based on the obtained results, SW480 cancer cell viability showed a significant decrease after 96 h of treatment with mumps M protein compared to that of the control. Approximately 76% of the cells died following treatment with pcDNA3-M after 48 h, whereas the percentage of cell death increased to 81.7% in the pcDNA3-M group 96 h after transfection.

### M protein of mumps virus increased apoptosis in SW480 cells over time

**Fig 2:**
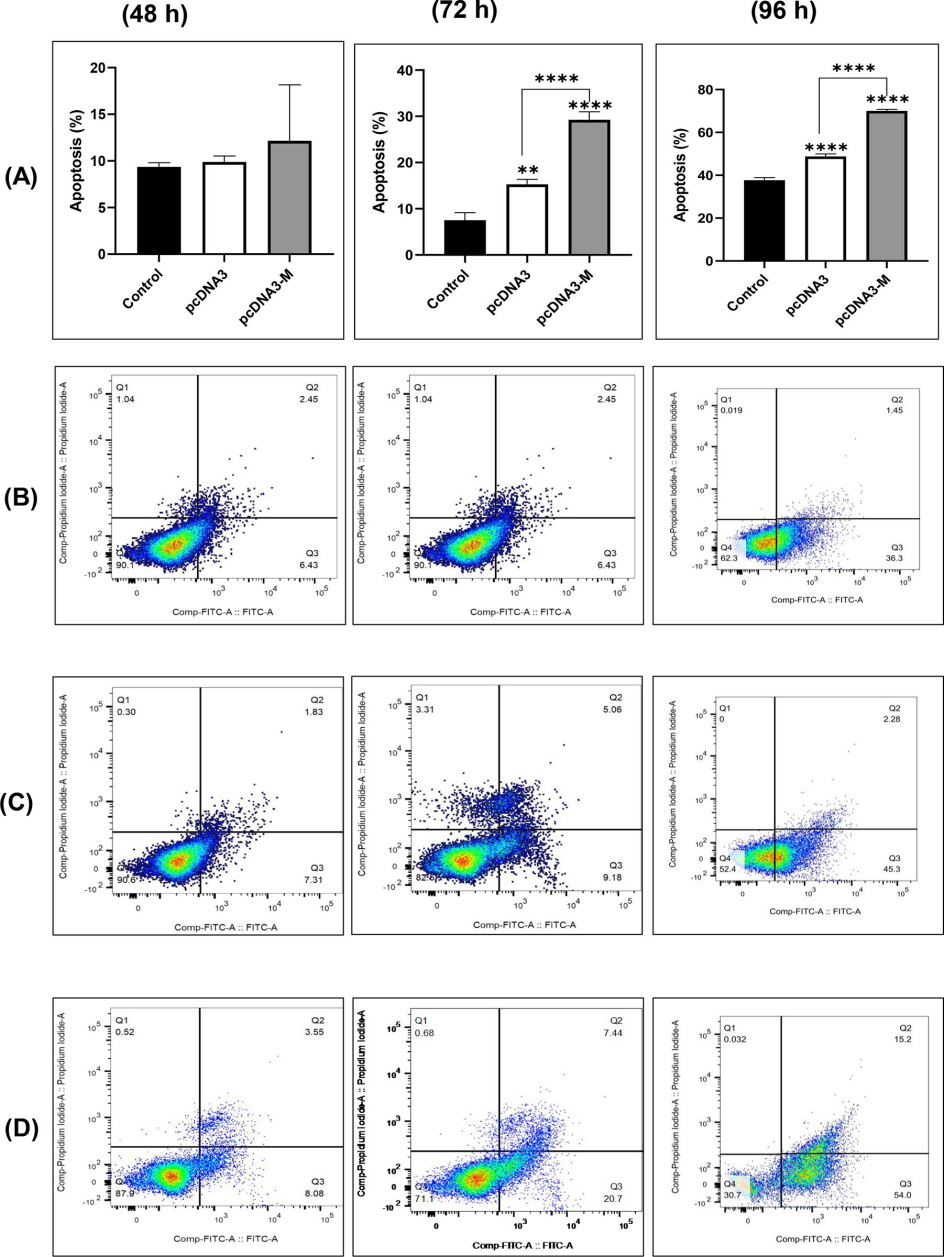
Evaluation of percentage of apoptotic SW480 cells after treatment. SW480 cells were transfected with pcDNA3 and pcDNA3 harboring the mumps virus M gene, and flow cytometry analysis was performed to compare the level of apoptosis between the groups. Apoptosis rates (%) at 48 h, 72h, and 96 h **(A)**. Flow cytometric dot plots of the control **(B)**, pcDNA3 **(C)**, and pcDNA3-M **(D)** in SW480 cells. The apoptosis rate in the pcDNA3-M group was significantly different from that in the control and vector groups at 72 and 96 h post-treatment and was time dependent. Distinct double-staining patterns detected the phases of apoptosis: viable (Annexin V- and PI-, lower left square), early apoptotic (Annexin V+ and PI-, lower right square), late apoptotic (Annexin V+ and PI+, upper right square), and necrotic cells (Annexin V- and PI+, upper left square). Data are presented as the mean ± standard deviation (n = 3). For all charts, **: *p<*0.01; ****: *p<*0.0001.

Flow cytometry was used to study the percentage of apoptosis of SW480 cancer cells after treatment with vector and pcDNA3-M groups in three time periods of 48, 72, and 96 hours. The flow cytometry analysis diagram of the control, vector, and pcDNA3-M was provided for SW480 cancer cells after 48 h (Fig 2). The percentages of apoptosis in SW480 cells after 48 h were 8.88, 9.14, and 11.63% in the control, vector, and pcDNA3-M cells, respectively. Statistical analysis of 3D images revealed no significant differences between the treated and control groups.

After 72 h, as shown in Fig 2, the percentage of cell death in the studied cells increased to 8.88, 14.24, and 28.14% in the control, vector, and pcDNA3-M groups,respectively. Statistical analysis (Fig 2) showed a significant increase in the percentage of apoptotic cells after treatment with pcDNA3 (*p<*0.01) and mumps *(p<*0.0001) compared to the control. Comparing the two treatment groups, the increase in the pcDNA3-M treatment group was more visible than that in the vector group *(p<*0.0001).

After 96 h, the percentage of cell death in the studied cells increased. In the control groups, the vector and pcDNA3-M were 37.75, 47.58, and 69.20%, respectively (Fig 2). Statistical analysis (Fig 2) showed a statistically significant increase in the percentage of apoptotic cells after treatment with the vector (*p<*0.0001) and pcDNA3-M (*p<*0.0001) compared to the control. The percentage of apoptotic cells increased in both the treatment groups. This was visible in the pcDNA3-M treatment group.This was also observed in the pcDNA3-M treatment group (*p<*0.0001). The highest apoptotic rate was observed in SW480 cells treated with pcDNA3-M for 96 hours.

### M gene of mumps virus upregulated the expression of apoptotic pathway proteins in SW480 cells

**Fig 3:**
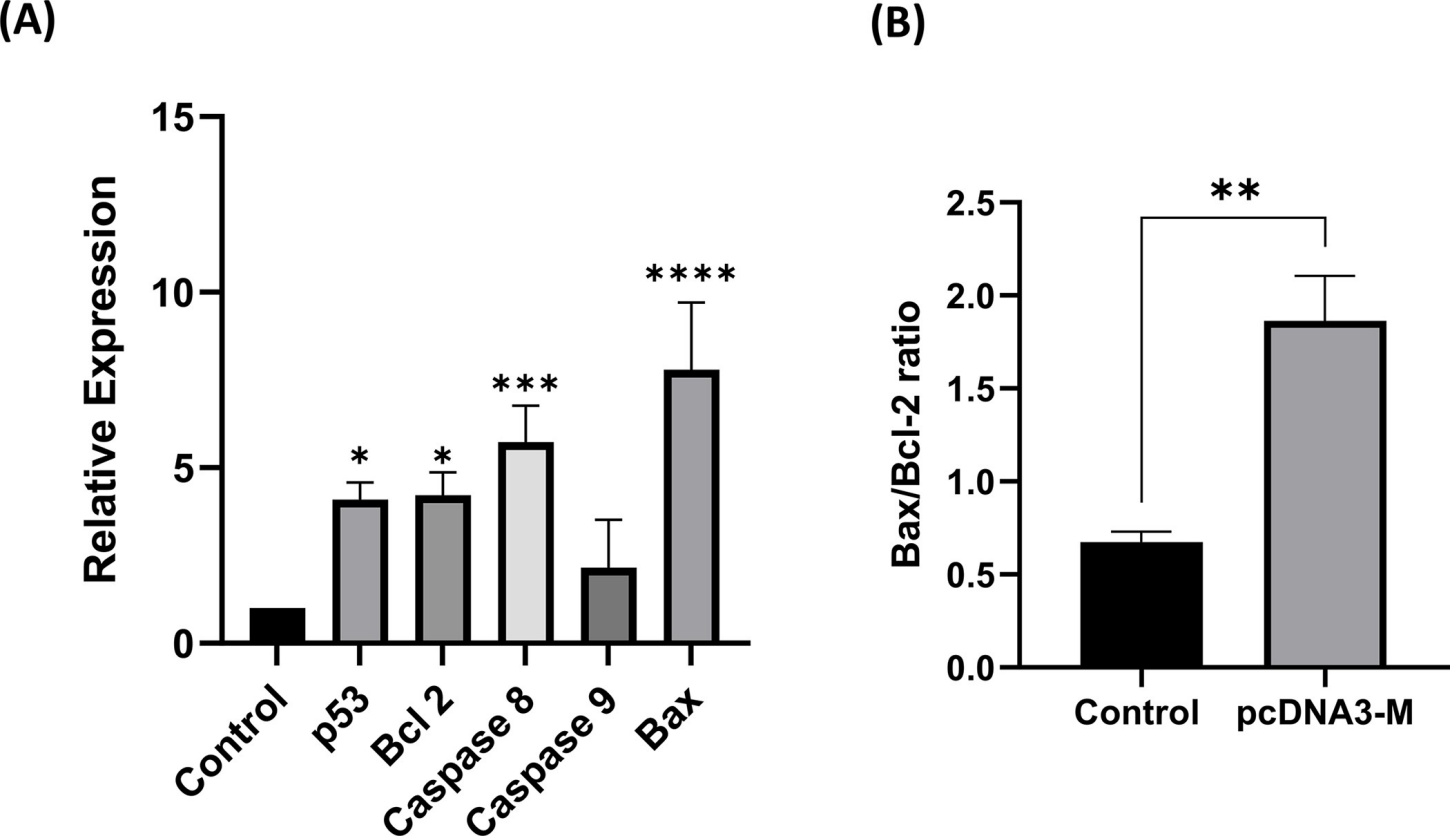
Role of apoptotic proteins in cell death induced by the mumps virus M gene. The relative mRNA levels of **(A)** Bax, Bcl2, caspase 8, caspase 9, and p53 and **(B)** the Bax/Bcl-2 ratio in SW480 cells treated with the mumps M gene were measured 72 h after treatment using real-time PCR. Each value was compared against the housekeeping gene GAPDH, which was set at a ratio of 1. Data are presented as the mean ± standard deviation (n = 3). For chart, *: *p<*0.05; **: *p<*0.01; ***: *p<*0.001; ****: *p<*0.0001.

qRT-PCR analysis was conducted to determine the relative expression of five specific genes (*Bax*, *Bcl-2*, *caspase 8*, *caspase 9*, and *p53*) in SW480 CRC cell lines. As per the results shown in Fig 3A, the p53 gene was found to be up-regulated in the sample group as compared to the control group by a mean factor of 4.072 (with an S.E. range of 3.622–4.597). Similarly, the Bcl-2 gene was expressed at a higher level in the sample group as compared to the control group by a mean factor of 4.156 (with an S.E. range of 3.599–4.897). The caspase 8 gene was significantly up-regulated in the sample group (compared to the control group) by a mean factor of 5.607 (with an S.E. range of 4.743–6.826). While the expression of caspase 9 was increased by more than two-fold by a mean factor of 2.382 (with an S.E. range of 1.707–3.395), there was no statistical difference compared to the control group (*p<*0.05). The Bax gene was expressed at a higher level in the sample group as compared to the control group by a mean factor of 7.629 (with an S.E. range of 5.951–9.783). Furthermore, the Bax/Bcl-2 ratio was observed to be 0.67 in control cells, whereas in the transfected cells, the ratio increased significantly to 1.86 (as shown in Fig 3B).

### Caspase 3 and caspase 7 activity increased over 27-fold in the m protein treated group compared to the control group

**Fig 4:**
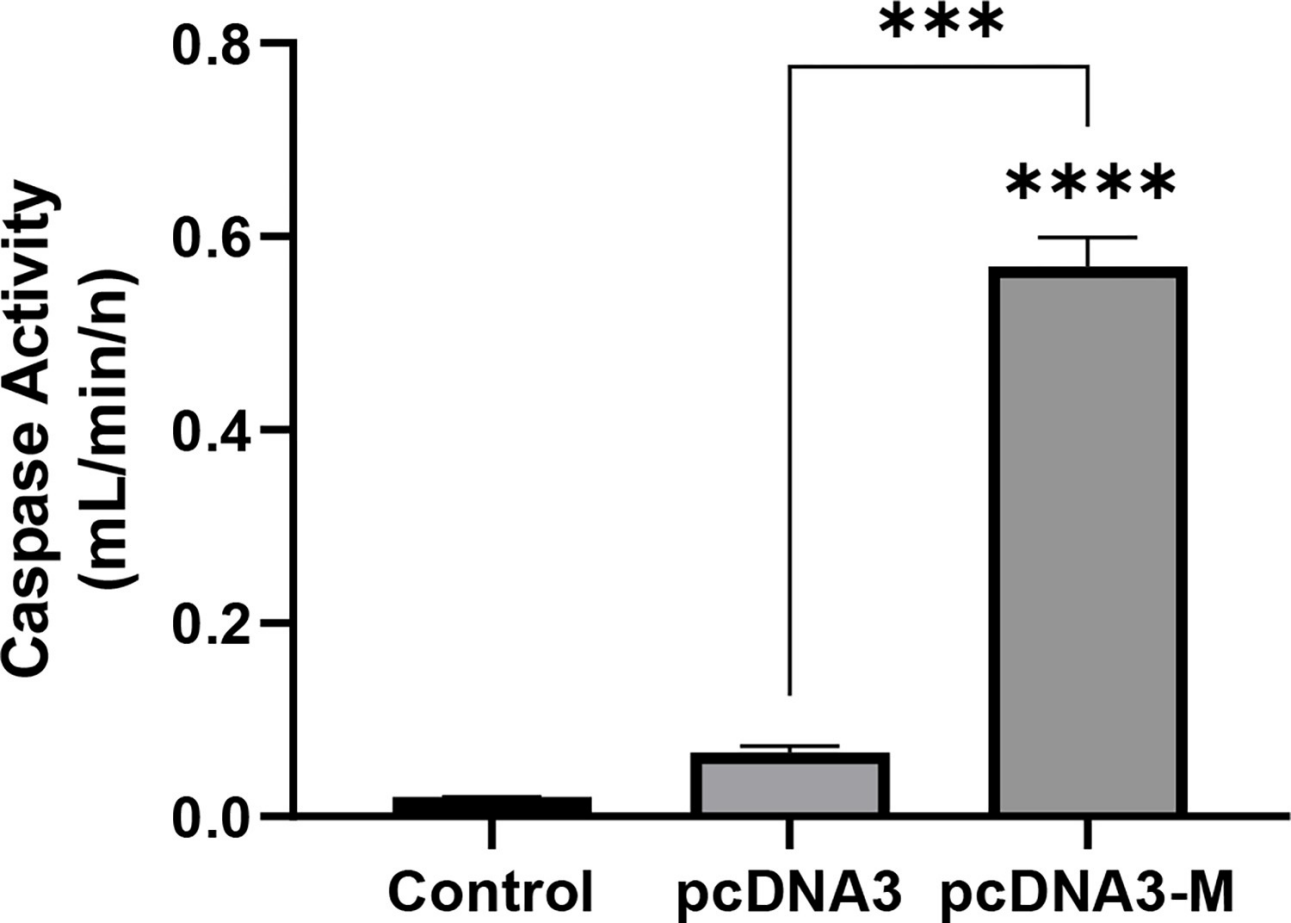
Role of caspase 3 and caspase 7 activity in SW480 cancer cell apoptosis induced by mumps M protein. The caspase 3 and caspase 7 activation rates (mL/min/n) in the mumps virus *M* protein group were determined 72 h post-treatment using the Caspase 3/7 Activity kit. Caspase activity in the experimental group was more than 28 times higher than that in the control group and 9 times higher than that in the vector group. Data are presented as mean ± standard deviation (n = 2). For chart, ***: *p<*0.001; ****: *p<*0.0001.

Caspases are intracellular protease enzymes that are involved in apoptosis and inflammation. Among these, caspases 3 and 7 play important roles in both internal and external apoptotic pathways. As shown in Fig 4, the increase in the activity of caspases 3 and 7 in the groups treated with the matrix of mumps virus was significant compared with that in the control (*p<*0.0001) and vector groups (*p<*0.001).

### BH3-like motif was detected in matrix protein of mumps virus

**Fig 5:**
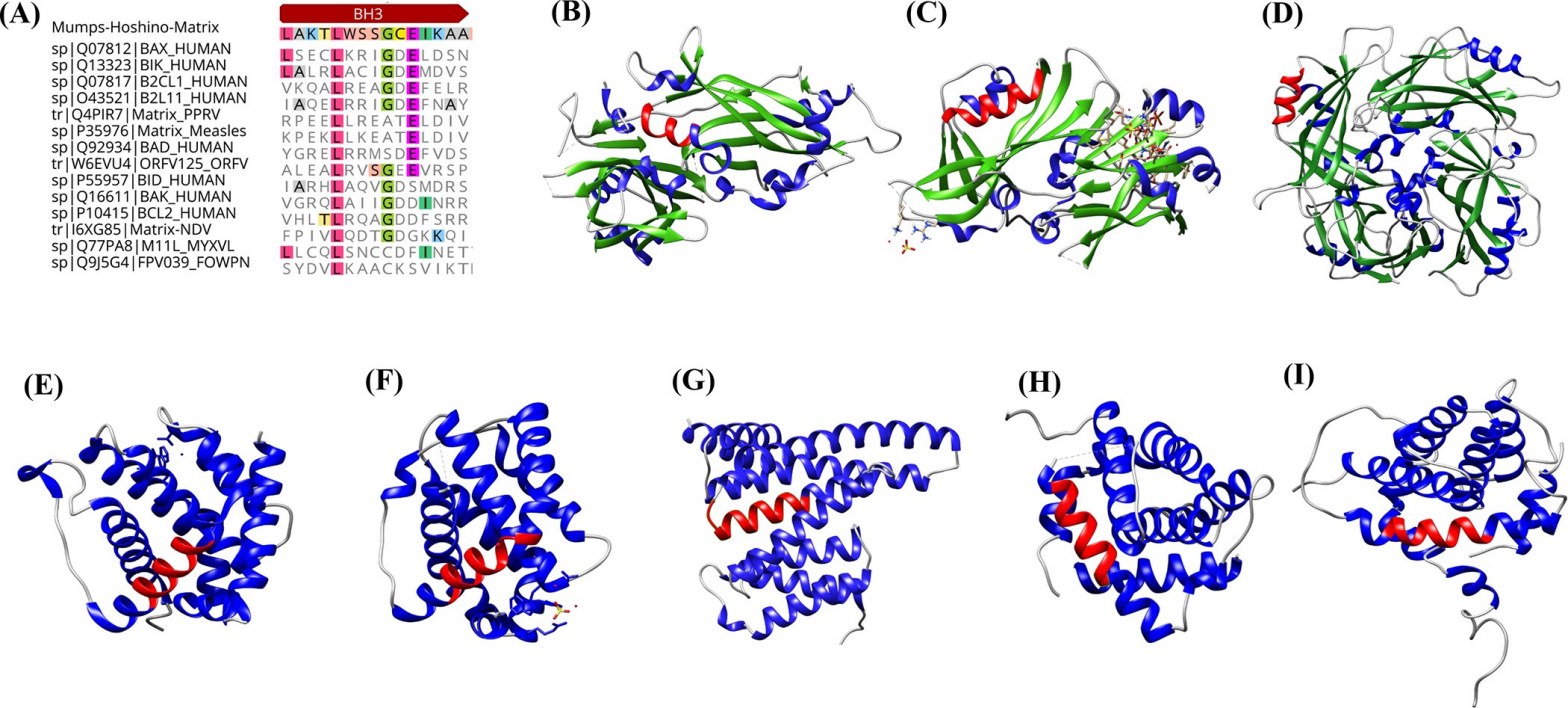
Alignment of the M protein of mumps virus with the BH3 motifs of cBcl2s and their viral homologs. **(A)**. Identical residues are highlighted. Some conserved residues in the BH3 motif of cBcl2 proteins, including leucine (L), glycine (G), and glutamic acid (E) were observed in the mumps-M protein (aa 312–326). The 3D structures of mumps-M **(B);** measles-M **(C);** PPRV-M **(D);** Bax **(E);** Bak **(F);** Bid **(G);** B2Cl1 **(H);** and Bcl2 **(I).** α-helices, β-sheets, and turns are shown in blue, green, and gray, respectively. Similar BH3 motif α-helix structures between mumps-M (aa 312–318) and Bcl-2 family proteins and their viral homologs are illustrated in red. Measles-M (aa 86–92); PPRV-M (aa 86–92); Bax (aa 59–65); Bak (aa 74–80); Bid (aa 86–92); B2Cl1 (aa 86–92); and Bcl2 (aa 93–99).

**Fig 6:**
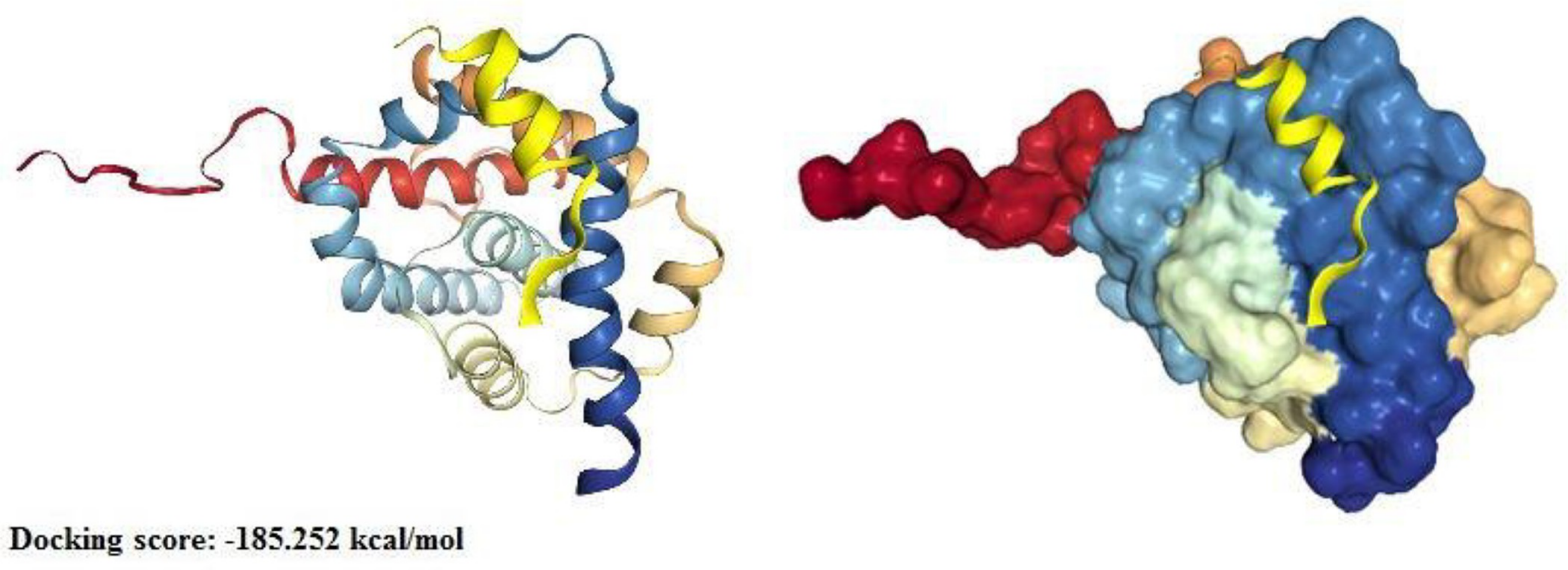
Potential interaction between the BH3-like motif of the mumps virus M protein and the Bax protein. The diagram illustrates the model with the highest score, indicating the most favorable binding between the two proteins.

**Fig 7:**
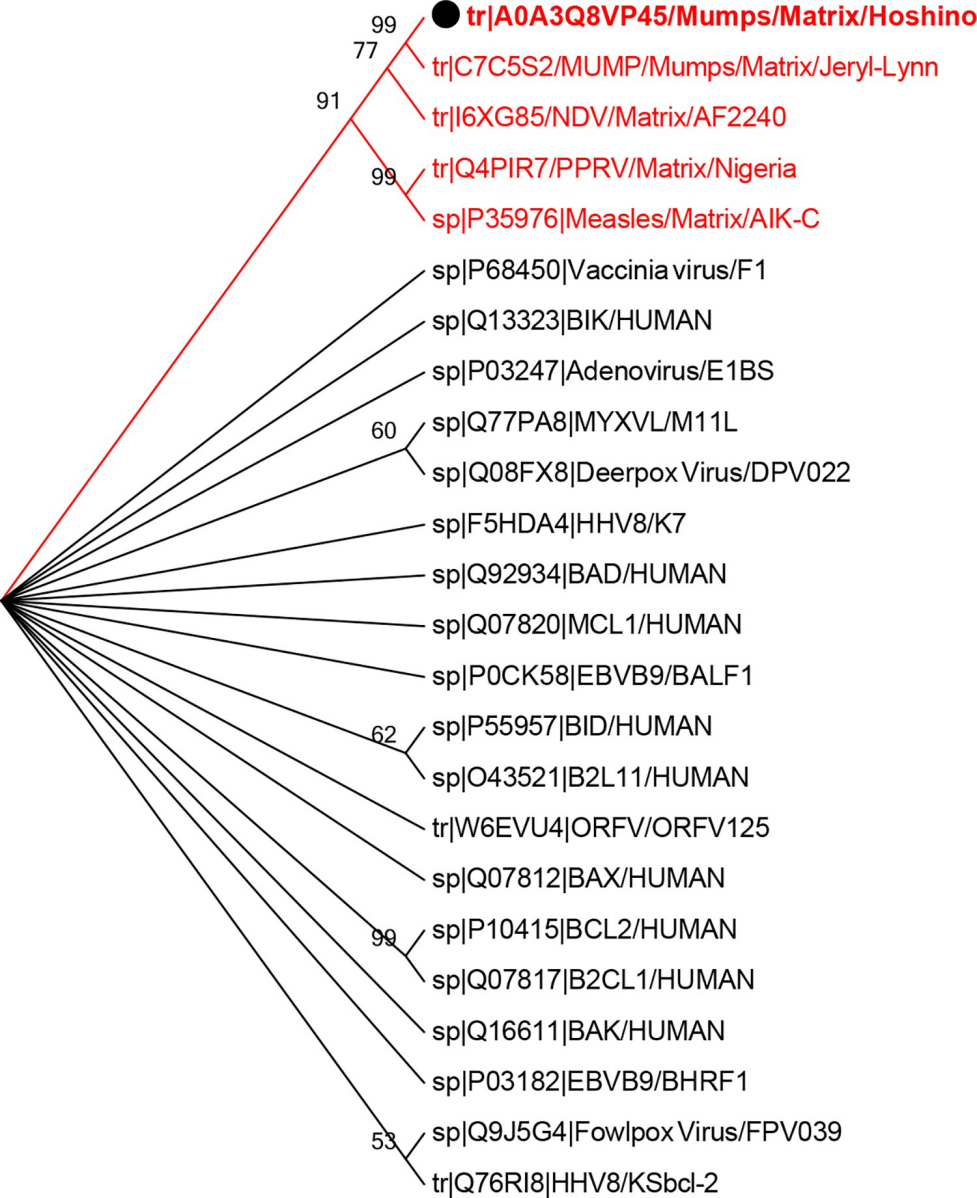
Phylogenetic tree of mumps virus M protein (bold sequence) and BH-containing proteins. A tree was generated using the neighbor-joining method. The M sequence under study had the highest similarity with paramyxovirus matrix proteins.

Sequence homology analysis revealed the presence of at least one BH motif (BH3) in mumps virus M protein (Fig 5). Moreover, the sequences between the studied matrix protein and matrix proteins of NDV (22.82%), measles virus (19.66%), and PPRV (18.52%) shared a higher overall identity than the other sequences, including Bcl-2 family proteins and their viral homologs. Some conserved residues within the BH domains of cBCl2 proteins, such as leucine (L), glycine (G), glutamic acid (E), phenylalanine (F), aspartic acid (D), asparagine (N), tryptophan (W), arginine (R), and valine (V), were detected at the same positions in the mumps-M sequence as well (Fig 5). However, because of their high gap frequency and low alignment score, the BH1, BH2, and BH4 motifs were not considered in further analysis. Additionally, we compared the M sequences of the Hoshino strain with those of other vaccine mumps virus strains, but no significant differences were observed between the strains. The closest sequences are shown in Fig 5. Indeed, the 3D structural analysis displayed a resemblance in the α-helix found within the BH3 motif (residues 312–318) of the M protein, similar to the α4-helixes present in Bax, Bid, Bcl-2, the α3-helix of B2Cl1, the α2-helix of Bak, and the α1-helixes of Bad. Similar α-helices were also detected in the BH3 motifs of M proteins from other paramyxoviruses, namely the measles virus and PPRV, except for the NDV AF2240 strain (Fig 6). Moreover, according to the findings from the docking analysis, the BH3-like motif of the matrix protein of the mumps virus has a potential interaction with residues 150–165 of the Bax protein, specifically recognized as the BH2 motif (Fig 7).

### A phylogenetic study showed evolutionary relationships between the matrix protein of the mumps virus and the matrix protein of other paramyxoviruses containing BH3 motif

A phylogenetic method was used to examine the evolutionary relationships among the M proteins from the paramyxovirus vaccine strains, Bcl-2 family members, and their viral homologs. This method placed the NDV matrix protein close to the mumps M protein (Fig 7). Additionally, the analysis indicated that the measles virus and PPRV, both belonging to the morbillivirus genus, were positioned next in terms of evolutionary similarity.

## Discussion

This study aimed to explore how the M protein of the mumps Hoshino vaccine strain affects the SW480 CRC cells. SW480 cells were transfected with a pcDNA3.1 Hygro(+) plasmid containing the mumps virus M gene. We investigated the effects of treatment using both bioinformatics and wet-lab experimental methods. The results revealed that transfected SW480 cells underwent apoptosis through the activation of apoptosis-mediated proteins.

Exploring viral death proteins may have clinical implications for tumor cell treatment. These proteins can be used against cancer cells by triggering apoptosis and inducing antitumor immunity. The roles of paramyxovirus proteins, including HN and M (NDV) [17,35,36], N and P (measles virus) [13,37], M (PPRV) [12], and V (mumps virus) [33], in apoptosis or survival of transformed cells have been examined. Although the oncolytic activity of the mumps virus has been previously validated in some studies [18,30,38–40], it has been shown that the mumps virus V protein modulates apoptosis in cervical adenocarcinoma cancer cells [16]. Hence, one question remains whether there is a relationship between tumor cell apoptosis and the expression of other viral proteins.

In this study, a marked increase in cell death was observed over time. According to the flow cytometry results, the pcDNA3-M treatment group showed the highest amount of apoptosis 96 h after transfection (70%) compared with the control and pcDNA3 groups. This increase was significant at 72 h and 96 h after transfection (*p<*0.0001). These results are consistent with a previous study on the M protein of vesicular stomatitis virus (VSV), which showed that it contributes to apoptosis in 63–68% of CRC cell lines [14]. Similar considerations have been applied to the M proteins of NDV and PPRV, highlighting their pro-apoptotic capabilities [12,17]. It is also interesting to indicate that the M protein immediately initiates the apoptosis process upon its entry into the cell. However, the detection of apoptosis as a cell pathway needs time to manifest. Therefore, the numbers of apoptotic cells between the treatment and control groups will likely be significant 72 hours after introducing the M protein into the cells.

In the MTT assay, we observed results that were comparable to those obtained using flow cytometry, although the cell viability levels were found to be lower. The disparity between both methods can be attributed to the different cell cultures employed in each technique. In particular, in the MTT assay, cells are cultivated in 96-well plates with a restricted surface area, while in flow cytometry, they are cultivated in larger 6-well plates. As such, the increased compaction and quicker cell death rates in MTT may result in decreased cell viability compared to flow cytometry.

The extrinsic signaling pathway is initiated upon death receptor-induced activation of caspase-8. Apoptosis may also be induced directly by BH3 domain–containing proapoptotic Bcl-2 family members in the intrinsic pathway. However, the cleavage of Bid to tBid by caspase 8 during death receptor-mediated apoptosis may trigger mitochondrial damage and activate the intrinsic apoptosis initiator protease caspase 9, as well. Both apoptotic pathways converge at one point, and effector caspases, including caspases 3 and 7, are activated following downstream apoptotic functions. There is considerable variation in the expression of pro-apoptotic factors in colon cancer [41–44]. Recent discoveries have revealed that the downregulation of caspase 3 and caspase 9 is associated with clinical manifestations and poor clinical outcomes in patients with CRC [29,44–46]. The results obtained from qPCR in the present study showed that SW480 cancer cells treated with the mumps virus M protein progressed to apoptosis by increasing the expression of the apoptosis proteins p53, caspase 8, and caspase 9. Although the expression of caspase 9 did not increase as much as that of other apoptotic proteins, the gene expression profile of SW480 cells, MTT, and flow cytometry results indicated that apoptosis was induced in cells treated with M protein. The higher expression level of caspase 8 than caspase 9 may be connected to other functions of caspase 8, particularly in cell adhesion and motility [44]. Indeed, the analysis of caspases 3 and 7 activity by ELISA in the current study revealed that these enzymes were 2800% and 900% more active in the pcDNA3-M treatment group than in the control and pcDNA3 groups, respectively. Furthermore, the M protein of the studied mumps virus strain changed the Bax/Bcl-2 ratio. In other studies, we found that the M protein of the measles virus (manuscript in preparation) and PPRV [12] from the *Paramyxoviridae* family, unlike the M protein of the Malaysian NDV strain [17], increased the Bax/Bcl-2 ratio in CRC cells. The Bax/Bcl-2 ratio is a key checkpoint that determines the outcome of cells toward apoptosis or survival. Performing a quantitative analysis, Raisova et al. suggested that a Bax/Bcl-2 ratio >1.00 accelerated apoptosis in melanoma cells, whereas an imbalance in this ratio increased the metastatic potential and resistance of tumor cells to death [47]. The expression system, type of cell line, and viral strain can change the protein expression pattern and the duration and rate of apoptosis [29,35,48–50]. For example, G protein of non-pathogenic rabies virusesactivates caspases 3, 8, and 9 in human lymphoblastoid and neuronal cell lines. Nevertheless, these cells do not undergo apoptosis after treatment with the G proteins of highly pathogenic strains [49]. Kopecky showed that apoptosis in BHK cells upon infection with M protein of recombinant wild-type VSV occurred more slowly than in HeLa cells [51].

The use of oncolytic viruses contributes to the mechanisms that have evolved over millions of years during the coexistence of humans and viruses [52,53]. Recent research has revealed that some viruses have BH regions that target mitochondrial membrane permeabilization directly or interact with certain Bcl-2 family proteins (Table 1). Molouki et al., discovered that redistribution of Bax to mitochondria and subsequent apoptosis of HeLa cells following M protein expression in NDV-infected cells occurred by BH3 domain of the protein [17]. Some viral proapoptotic proteins, such as HBx from hepatitis B virus (HBV) [54], Vpr from human immunodeficiency virus-1 (HIV-1), matrix protein from PPRV [12], and PBF1-F2 from Influenza A virus (IAV) [55] exhibit α-helical structures that are integral to their proapoptotic function and exert potential pore-forming capabilities. In contrast, homologs of cellular anti-apoptotic Bcl-2 proteins have also been detected in some DNA viruses such as adenoviruses, herpesviruses, and poxviruses, which protect host cells from premature death [46]. While the anti-apoptotic homologs of Bcl-2 can serve as a supportive response of DNA viruses to host cell immunity, facilitating their replication within host cells [46], apoptosis can also be employed as a strategy by certain viruses to promote their proliferation and survival within host cells. This mechanism allows the virus to exploit the breakdown of cellular structures, thereby increasing the secretion of essential nutrients that contribute to viral dissemination [56].

**Table 1:** Viral Bcl-2 homologs and their effect on the intrinsic apoptotic pathway.

Virus Name	Viral Protein	Viral domain	Function	Cellular Protein Target	Reference
**Adenovirus**	E1B-19k	BH1,BH3	Anti-apoptotic	Bax/Bak	[21]
**NDV**	M protein	BH3	Pro-apoptotic	Bax	[17]
**PPRV**	M protein	BH3	Pro-apoptotic	Bax	[12]
**HBV**	HBSP	BH3	Pro-apoptotic	Bcl-2/Bcl-xl	[57]
**Myxoma Virus**	M11L	BH3	Anti-apoptotic	Bax, Bak	[56,58]
**HCV**	Core protein	BH3	Pro-apoptotic	Mcl-1	[59,60]
** EBV**	BHRF1	BH1, BH2, BH3,BH4	Anti-apoptotic	BIM, BID, PUMA, BAK	[61]
**Vaccinia virus**	F1L	BH3	Anti-apoptotic	Bak, Bim, Bax	[62]
**Orf Virus**	ORFV125	BH3	Anti-apoptotic	Bik,Puma,Noxa, Bim	[63]
**Fowlpox Virus**	FPV039	BH1, BH2, BH3,BH4	Anti-apoptotic	Bax, Bak, BimL, Bik	[64,65]
**Herpesvirus Saimiri**	ORF16	BH1, BH2	Anti-apoptotic	Bax, Bak	[23]
**KSHV**	K7	BH2	Anti-apoptotic	Bcl-2	[66]
**CMV**	vMIA	BH3	Anti-apoptotic	ANT, Bax	[67–69]
** ASFV**	A179L	BH1	Anti-apoptotic	Bid, Bax, Bak, Noxa	[70]
**Mumps Virus**	M protein	BH3	Pro-apoptotic	Bax	This study

*Abbreviations: NDV (Newcastle Disease Virus), PPRV (Peste des petits ruminants virus), HBV (Hepatitis B virus), HCV (Hepatitis C virus), EBV (Epstein-Barr Virus), KSHV (Kaposi Sarcoma-Associated Virus), CMV (Cytomegalovirus), ASFV (African swine fever virus)

In agreement with these studies, alignment of the mumps M protein with members of the Bcl-2 family and their paramyxovirus homologs indicated that the M protein contains expected alpha structural characteristics and important functional residues with Bcl-2 proteins and M proteins of other paramyxoviruses, such as NDV, PPRV, and measles virus. This resemblance approximates the BH3 domain (Fig 5). The BH3 domain, which is found in both pro-apoptotic and anti-apoptotic members of the Bcl-2 protein family, can mediate the interaction between these two groups involved in regulating cell death pathways [21]. Furthermore, the interaction between the BH3 region in one protein and the hydrophobic pocket in the other protein formed by BH1 to BH3 can either suppress or enhance mitochondrial membrane permeabilization [15]. The data for the mumps virus provided in this article indicate a significant role for Bax protein as an M target. It is possible that the M protein acts as a receptor for the BH domains of the core pro-apoptotic Bcl-2 family proteins,namely, Bax, and improves their function. Docking analysis indicated that the M protein effectively attaches to the BH2 motif of Bax via its BH3 motif, consequently inducing Bax activation (Fig 6). Following this activation, Bax protein on the mitochondrial membrane is stimulated to oligomerize, ultimately triggering the intrinsic apoptotic process. However, whether the M protein mainly targets the core apoptotic protein rather than its upstream BH3 activators has yet to be determined.

**Fig 8:**
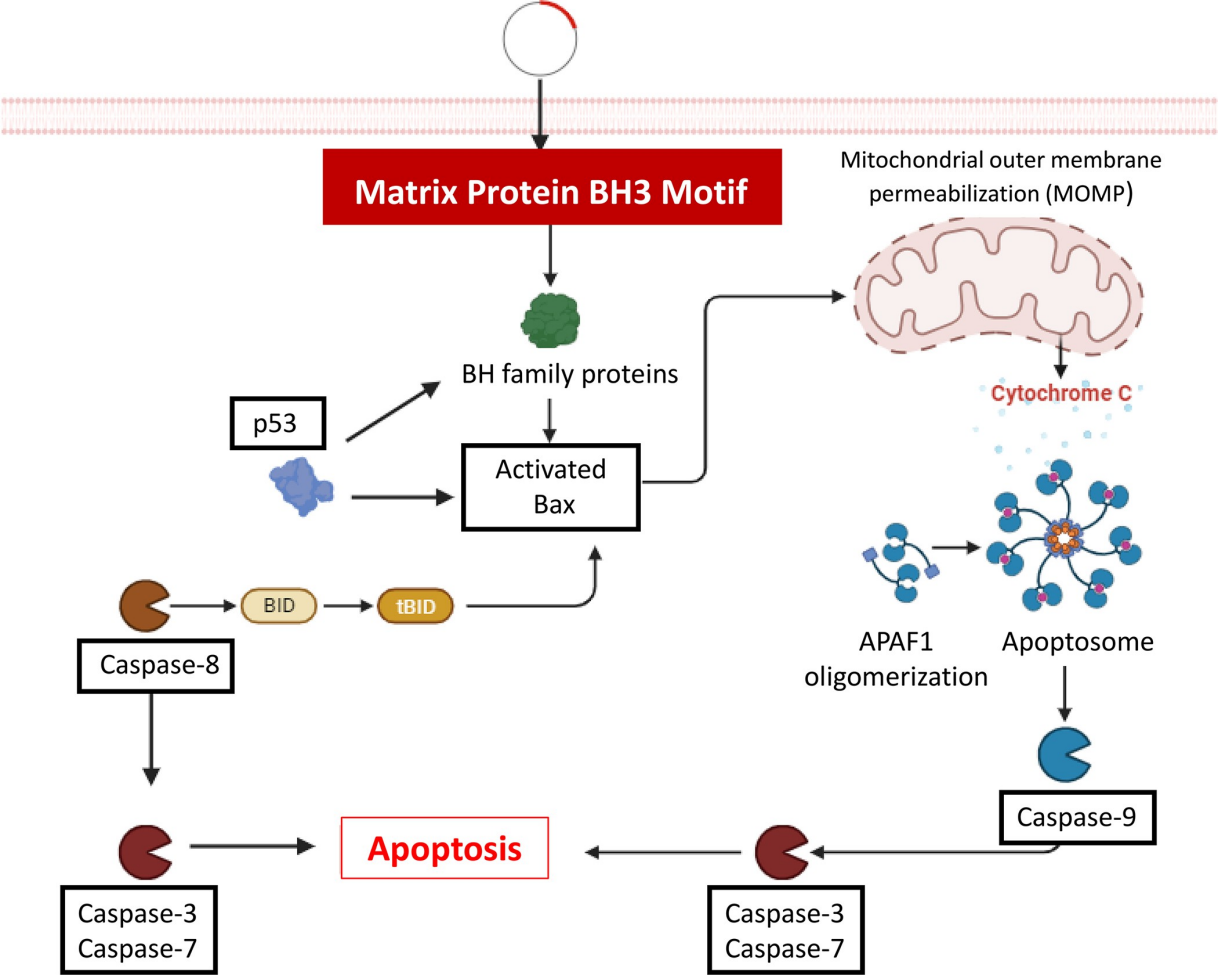
Schematic of the proposed role of the matrix protein of mumps virus in apoptosis. The matrix protein may activate different proteins involved in the intrinsic apoptotic pathway, including Bax, through its BH3 motif. Once activated, Bax undergoes structural changes, creating oligomers that cause the permeabilization of the outer mitochondrial membrane (OMM). In turn, this event activates the caspase cascade in the host cell, contributing to the progression of apoptosis. During death receptor-mediated apoptosis, the cleavage of Bid to tBid by caspase 8 may trigger mitochondrial damage and activate the intrinsic apoptosis initiator protease caspase 9. The proteins upregulated in this study are highlighted in the black boxes. The figure was created with the scientific image and illustration software https://biorender.com.

Although low sequence identity was observed over a 375-amino acid stretch of the M protein compared to that of cBcl-2s, this lack of homology between viral and host proteins could not rule out their structural or functional homology [22,46]. According to the sequence investigation and phylogenetic study, the M protein of the mumps virus showed a similar relationship to the M sequence of the NDV Malaysian strain AF2240 (genus Orthoavulavirus), followed by the PPRV and measles virus vaccine strains (genus Morbillivirus) (Fig 7). This may be because of their similar evolutionary and functional properties. In this respect, it has been demonstrated that AF2240-M directly binds to Bax protein via its BH3 domain and enhances Bax redistribution to the mitochondria. The interaction of PPRV [12] and measles virus (manuscript in preparation) M proteins with Bax and stimulation of the intrinsic apoptosis pathway have also been approved in our previous research. The sequence and functional homology between the mumps virus and other paramyxoviruses may indicate that the mumps virus M protein is another viral Bcl-2 homolog. Although a precise conclusion regarding the role of BH domains requires further investigation using different mutants of the virus and additional cell lines, the overall findings confirm the apoptotic characteristics of the mumps M protein, which could be achieved through interaction with the upregulated Bax protein and induction of the intrinsic apoptotic pathway. The hypothesized role of the BH3 motif of the virus is illustrated in the Fig 8.

## Conclusions

The findings of this study suggest that there may be a connection between the oncolytic activity of the M protein of the Hoshino commercial vaccine strain of the mumps virus and apoptosis in colorectal cancer cells. The M protein was found to increase the Bax/Bcl-2 ratio, activate pro-apoptotic proteins, and induce apoptosis by interacting with the host Bcl-2 protein, specifically Bax, through its BH3 domain. Therefore, M protein alone or in combination with other therapeutic approaches could be a promising target for cancer treatment. However, further research is needed to understand the underlying mechanism by which the mumps virus triggers cell death.

## Supporting information

S1 Fig (PNG)

S1 Table OD and normalize of viability of MTT assay 48 h,72 h, and 96 h post-transfection. (XLSX)

S2 Table Percentage of live cells, early apoptosis, late apoptosis, and necrosis at different time points (48 h, 72 h, and 96 h) in the flow cytometry assay. (XLSX)

S3 Table Relative expression report of qPCR 72 h post-transfection. (DOC)

S4 Table Results of ELISA-assessed activities of caspases 3 and 7, 72 h post-infection. (XLSX)
