# Supplementary material for: The power of mumps virus: Matrix protein activates apoptotic pathways in human colorectal cell lines
Source: PLoS One. 2023 Dec 13;18(12):e0295819. doi: 10.1371/journal.pone.0295819 (PMC10718445; doi:10.1371/journal.pone.0295819)
Supplement: S3 Table — (DOC) [file pone.0295819.s004.doc]

# S3 Table. Relative expression report of qPCR 72 post-infection.

| **Gene** | **Type** | **Reaction Efficiency** | **Expression** | **Std. Error** | **95% C.I.** |
| --- | --- | --- | --- | --- | --- |
| GAPDH | REF | 0.7875 | 1.000 |  |  |
| P53 | TRG | 0.7275 | 4.072 | 3.622 - 4.597 | 3.433 - 4.836 |
| Bcl2 | TRG | 0.71 | 4.156 | 3.599 - 4.897 | 3.186 - 5.453 |
| Caspase 9 | TRG | 0.7375 | 2.382 | 1.707 - 3.395 | 1.506 - 3.790 |
| Caspase 8 | TRG | 0.7375 | 5.607 | 4.743 - 6.826 | 4.090 - 7.750 |
| Bax | TRG | 0.6325 | 7.629 | 5.951 - 9.783 | 5.864 - 9.926 |

Legend:

*TRG - Target*
*REF - Reference*

|  |
| --- |
